# Survival analysis using machine learning in transplantation: a practical introduction

**DOI:** 10.1186/s12911-025-02951-7

**Published:** 2025-03-21

**Authors:** Andrea Garcia-Lopez, Maritza Jiménez-Gómez, Andrea Gomez-Montero, Juan Camilo Gonzalez-Sierra, Santiago Cabas, Fernando Giron-Luque

**Affiliations:** 1 Research Department, Colombiana de Trasplantes, Bogotá, Colombia; 2 Universidad de los Andes, Bogotá, Colombia; 3 Transplant Surgery Department, Colombiana de Trasplantes, Bogotá, Colombia

**Keywords:** Transplantation, Machine Learning, Artificial Intelligence, Survival Analysis

## Abstract

**Background:**

Survival analysis is a critical tool in transplantation studies. The integration of machine learning techniques, particularly the Random Survival Forest (RSF) model, offers potential enhancements to predictive modeling and decision-making. This study aims to provide an introduction to the application of the RSF model in survival analysis in kidney transplantation alongside a practical guide to develop and evaluate predictive algorithms.

**Methods:**

We employed a RSF model to analyze a simulated dataset of kidney transplant recipients. The data were split into training, validation, and test sets using split sample (70%-30%) and cross-validation (5-folds) techniques to evaluate model performance. Hyperparameter tuning strategies were employed to select the best model. The concordance index (C-index) and Integrated Brier Score (IBS) were used for internal validation. Additionally, time-dependent AUC, F1 score, accuracy, and precision were evaluated to provide a comprehensive assessment of the model's predictive performance. Finally, a Cox Proportional Hazards model was fitted to compare the results of the main metrics between both models. All analyses were supported by step-by-step code to ensure reproducibility.

Findings.

The RSF model obtained a C-index of 0.774, an IBS of 0.090. The F1 score was of 0.945, accuracy was 89.67 and precision was 90.99%. The time-dependent ROC analysis produced an AUC of 0.709, indicating a moderate predictive performance. Lastly, the analysis shows that the three most important variables are donor age, BMI, and recipient age.

**Conclusions:**

This study demonstrates the robustness and potential of the RSF model in kidney transplant analysis, achieving strong validation metrics and highlighting its advantages in managing complex, censored data, while emphasizing the need for further exploration of hybrid models and clinical integration.

**Supplementary Information:**

The online version contains supplementary material available at 10.1186/s12911-025-02951-7.

## Introduction

### Background

Kidney transplantation significantly enhances the quality of life for patients with end-stage renal disease [1, 2]. Timely and precise estimates enable healthcare providers to optimize patient management, allocate resources efficiently, and improve patient outcomes. In transplantation research, the primary focus is often on survival outcomes, as understanding and predicting patient survival is essential for advancing clinical decision-making and patient care. Accurate survival predictions can significantly enhance transplant outcomes by identifying high-risk patients, optimizing donor-recipient matching, and tailoring post-transplant care [1].

To date, most clinical prediction models for survival in transplantation have relied on the Cox proportional hazards regression model [2]. This approach has been widely utilized to estimate the risk of graft loss or patient death by analyzing the relationship between time-to-event outcomes and relevant covariates. Tools like the iBox score, a widely used and validated predictive model across multiple cohorts, have enhanced predictive accuracy by integrating clinical, histological, and immunological factors [3]. However, the Cox model has notable limitations, including its reliance on strict assumptions regarding covariates, challenges in managing large datasets, susceptibility to instability when dealing with highly correlated covariates, handling of missing data and non-linear relationships between variables [4].

Recent advances in artificial intelligence (AI) and machine learning (ML) have provided new tools for improving transplantation decisions [2]. One example is the Random Survival Forest (RSF), a method used for survival analysis that, unlike the Cox model, can handle highly correlated variables and does not depend on strict data assumptions [3]. A 2019 systematic review of kidney transplantation predictive models identified artificial neural networks, decision trees, and Bayesian belief networks as the most common ML methods, although only one model accounted for time-to-event (survival) data [1]. Similarly, a 2022 systematic review of lung transplantation reported that 25% of studies used Random Forest models to predict clinical and quality-of-life outcomes, but none applied the RSF model [4]. Additionally, a recent meta-analysis indicates that ML models often outperform traditional approaches, achieving better diagnostic accuracy, sensitivity, specificity, and area under the receiver operating characteristic curve (AUC-ROC) values [5].

Nevertheless, there is evidence that traditional prognostication systems, such as those based on the Cox model, can be as effective as ML methods in certain circumstances [6–8]. This discrepancy indicates that ML does not always provide a definitive advantage in highly complex or high-dimensional settings, possibly due to variations in study design, data quality, or model implementation.

Consequently, hybrid approaches have emerged to harness the strengths of both classical and ML-based models. These methods combine Cox regression with RSF or neural networks to capture linear, non-linear, and complex relationships more comprehensively [9]. In addition, ensembles of ML methods, including Neural Random Forest and Gradient Boosting Machines, are increasingly applied to enhance predictive accuracy further [10]. By integrating the advantages of both traditional and ML-driven strategies, researchers can gain deeper insights into post-transplant outcomes while also identifying the specific conditions under which ML models offer meaningful improvements in clinical decision-making and patient care.

### What this paper will achieve

This study aims to explore the application of the RSF model in the context of kidney transplantation. By leveraging a simulated dataset of kidney transplant recipients, we demonstrate the practical implementation of the RSF model for survival analysis. Our approach includes a detailed guide to developing and evaluating predictive algorithms, ensuring reproducibility through step-by-step code.

### How to follow this paper

This paper offers a structured, step-by-step guide to performing survival analysis using RSF. We begin with a conceptual overview and proceed to practical instructions in the R Statistical Programming Environment. The first section introduces the typical stages of a ML analysis, while the second section provides illustrative R code for running these analyses. Designed as an educational resource, this work aims to help researchers and clinicians apply RSF models in survival analysis. By offering clear explanations and reproducible code, we seek to make ML methodologies more accessible in the field of kidney transplantation research.

## Methods

### Dataset

The dataset utilized in this study comprises information on patients who have undergone kidney transplants. This dataset was generated using simulated data, with the corresponding code available in *Supplementary Material 1*. The generated dataset itself can be found in *Supplementary Material 2*, and the R script used for the analysis is provided in *Supplementary Material 3.* The variables included in the dataset are well-supported by clinical evidence and research literature as critical predictors of kidney graft outcomes. Recipient age is included as older patients face higher risks of graft loss due to comorbidities and weaker immune responses [11–13]. Body Mass Index (BMI) is relevant because obesity can increase surgical risks and place extra stress on the kidney [12, 14]. The presence of diabetes and hypertension in recipients is coded as 0 (no) or 1 (yes), as both conditions can directly harm graft function through complications like vascular damage and diabetic nephropathy [11, 12]. Donor age, coded as 0 for donors under 60 years and 1 for those 60 or older, is considered because older kidneys are more prone to injury and reduced function [11, 15]. Cold ischemia time, measured in hours, is included because longer preservation can cause ischemic damage to the graft [13, 16]. Finally, the time from transplant to graft loss (in years) and an indicator of whether graft loss occurred (coded as 0 for no and 1 for yes) are critical for evaluating transplant success [12, 13].

This dataset is utilized to analyze the performance of the RSF model in predicting kidney graft loss, where the event of interest is the loss of the kidney graft, and the time variable is the duration from the transplant to the occurrence of the graft loss. Unlike public databases, which contain real-world data, simulation allowed us to control the characteristics of the variables and avoid common issues such as missing data or inconsistencies. However, this decision carries implications that will be discussed later.

## Software

R version 4.4.3 was used for all analyses. The practical application of the code has been designed to run relatively quickly on a personal device.

### Conducting random survival forest analysis

The following section will take you through the necessary steps of an RSF analysis using the Transplant dataset.

1. Importing and preparing the dataset
2. Data splitting
3. Training the RSF algorithm
4. Testing the RSF algorithm
5. Model validation: Assessing performance of RSF algorithm
6. Plotting variable importance
7. Comparison to Cox Hazard Model

### Step 1: Importing and preparing the dataset

The dataset, *transplant_database.xlsx*, was imported using the *readx*l library in R. The following code shows how the dataset was read into a data frame named* data* for analysis.

#### Code Block: Importing the dataset

# Load the required library.

library(readxl).

# Import the dataset from an Excel file.

transplant_database <—read_excel("transplant_database.xlsx").

# Assign the dataset to a variable.

data <—transplant_database.

**Fig. 1:**
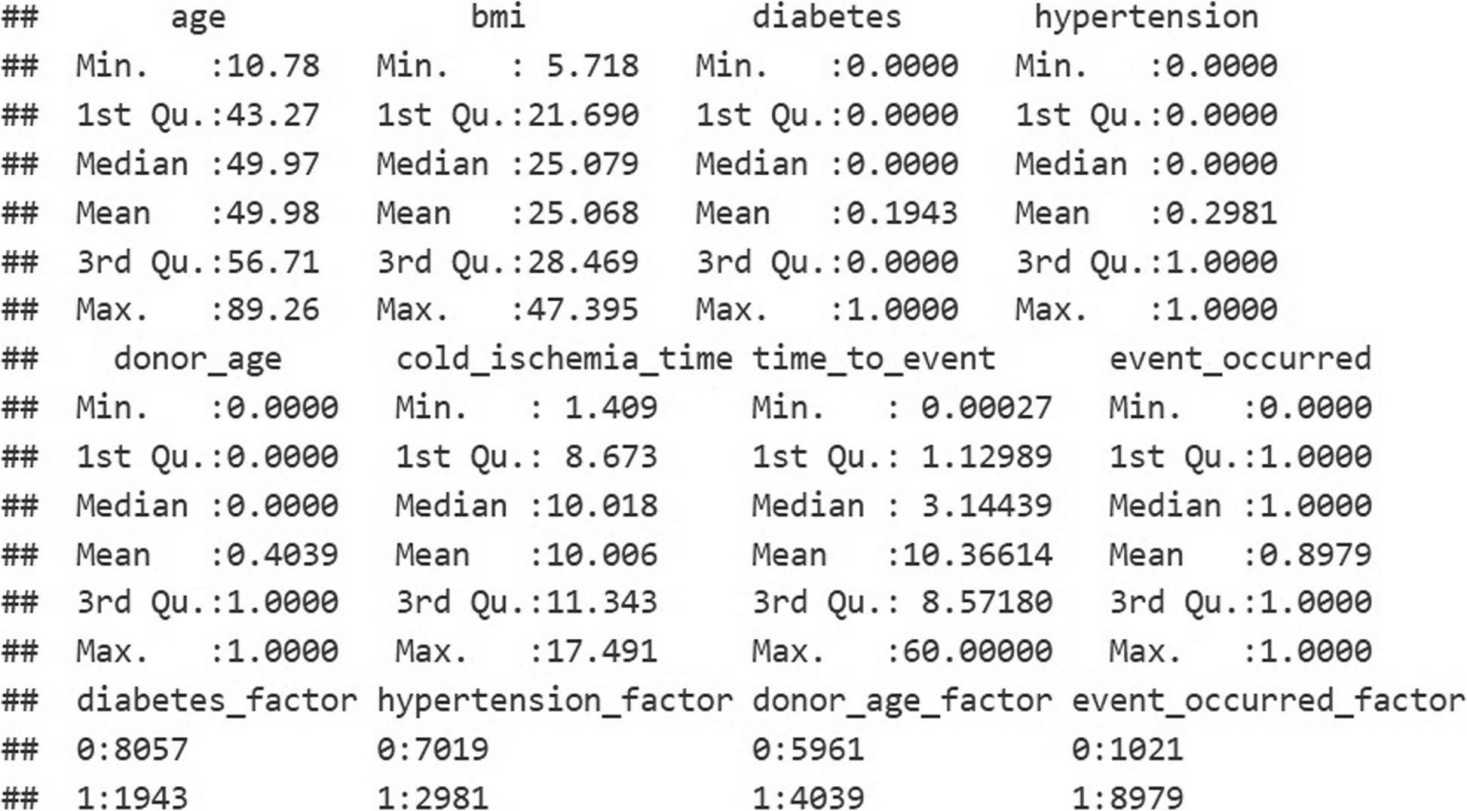
Data exploration. Summary of descriptive statistics for the simulated kidney transplant dataset. Variables include recipient age, body mass index (BMI), donor age, cold ischemia time, and graft survival time. The figure provides a clear overview of minimum, maximum, mean, median, and interquartile ranges for these variables, which are key for understanding the dataset’s characteristics

An initial exploration was conducted with *summary(data)* shown in Fig. 1. The initial exploration revealed a total of 10,000 observations and 8 variables. The statistical summary reported frequencies, ranges, means, and the presence of missing values, which are explicitly reported when detected. We identified a total of 8979 events that occurred.

### Step 2: Data Splitting

Data splitting is an essential practice in predictive analysis [17]. This step involves splitting the data into training, validation and test sets, typically in a 70–30 ratio. This split enables the assessment of the model's predictive performance on unseen data, ensuring robust generalization to new data [18].

The code is used to split a dataset into training and testing sets for model evaluation. The *set.seed[42]* function ensures reproducibility of results by setting a random seed. The variable *n* captures the number of rows in the dataset, and the *sample* function randomly selects 70% of indices to create the training set *train_data*. The remaining 30% is assigned to the testing set *test_data*. This process allows training on one subset and testing on another to assess performance. The following R code demonstrates this approach:

#### Code Block: Splitting the dataset into training and testing sets

# Set a random seed for reproducibility.

set.seed[42].

# Split the data into training (70%) and testing (30%) sets.

n <—nrow(data).

train_indices <—sample(1:n, size = 0.7 * n).

train_data <—data[train_indices,]

test_data <—data[-train_indices,]

### Step 3: Training the RSF algorithm

The RSF is an extension of the popular Random Forest algorithm, specifically designed for survival analysis to predict the time until an event occurs (e.g., transplantation, relapse, or death) [19]. Unlike traditional parametric models, RSF does not rely on assumptions, making it particularly useful for handling nearly correlated variables and large datasets in real-world scenarios.

RSF operates by creating nodes where the risk of an outcome is assessed based on the influencing variables. At each node, a random subset of the data is analyzed, and the average risk of the event occurring is calculated for the entire population [20].

To develop and refine the RSF model for time-to-event data, a five-fold cross-validation scheme was defined using the *trainControl* function from the *caret* package (*method = "cv", number = 5*), ensuring that each observation would serve as validation data exactly once. Concurrently, a grid of potential hyperparameter values was created with *expand.grid*, specifying different combinations for the number of trees (*ntree*), the minimum size of terminal nodes (*nodesize*), and the number of variables to consider at each split (*mtry*).

To streamline model training for each hyperparameter combination, a custom function named *train_rsf* was written. Internally, this function calls *rfsrc* from the *randomForestSRC* package, specifying parameters such as the survival formula (*Surv(time_to_event, event_occurred) ~ ...*), the number of random splits (*nsplit*), and variable importance tracking (*importance*). A fixed random seed (*seed = 42*) was also set for reproducibility.

Next, an iterative cross-validation was performed using *lapply*. For every row in the hyperparameter grid (*tune_grid*), the *train_rsf* function produced an RSF model, which was then evaluated on the test dataset via the *predict* function. The *concordance.index* function measured the model’s performance by calculating a concordance index (C-index), reflecting how accurately the model predicted the order of events. All models and their corresponding C-indices were collected in *cv_results*.

Finally, the configuration yielding the highest C-index was extracted from *cv_results* using a simple maximum search with *which.max*. The selected model, denoted by *best_model*, was printed to reveal its details (including sample size, number of trees, terminal node size, out-of-bag (OOB) scores, and so forth). The model was then re-fitted with these optimal hyperparameters to finalize the training procedure. The following code specifies this setup:

#### Code Block: Training the RSF model

# Define the cross-validation method.

cv_control <—trainControl(method = "cv", number = 5).

# Define the grid of hyperparameters to tune.

tune_grid <—expand.grid(ntree = c[50, 100],

nodesize = c(10, 15),

mtry = c(2, 3)).

# Function to train the model with specific hyperparameters.

train_rsf <—function(ntree, nodesize, mtry) {

rfsrc(*Surv(time_to_event, event_occurred)* ~ age + bmi + diabetes + hypertension + 

donor_age + cold_ischemia_time,

data = train_data,

ntree = ntree,

nodesize = nodesize,

mtry = mtry,

nsplit = 5,

importance = TRUE,

seed = 42).

}

# Perform cross-validation manually.

cv_results <—lapply(1:nrow(tune_grid), function(i) {

params <—tune_grid[i,]

model <—train_rsf(params$ntree, params$nodesize, params$mtry).

pred <—predict(model, newdata = test_data).

c_index <—concordance.index(pred$predicted, test_data$time_to_event, test_data$event_occurred).

list(model = model, c_index = c_index$c.index).

})

# Select the best model based on C-index.

best_model <—cv_results[[which.max(sapply(cv_results, function(x) x$c_index))]]$model.

print(best_model).

# Fit the selected best model using the training data.

best_model <—rfsrc(*Surv(time_to_event, event_occurred)* ~ age + bmi + diabetes + hypertension + 

donor_age + cold_ischemia_time,

data = train_data,

ntree = 100,

nodesize = 10,

mtry = 2,

nsplit = 5,

importance = TRUE,

seed = 42).

**Fig. 2:**
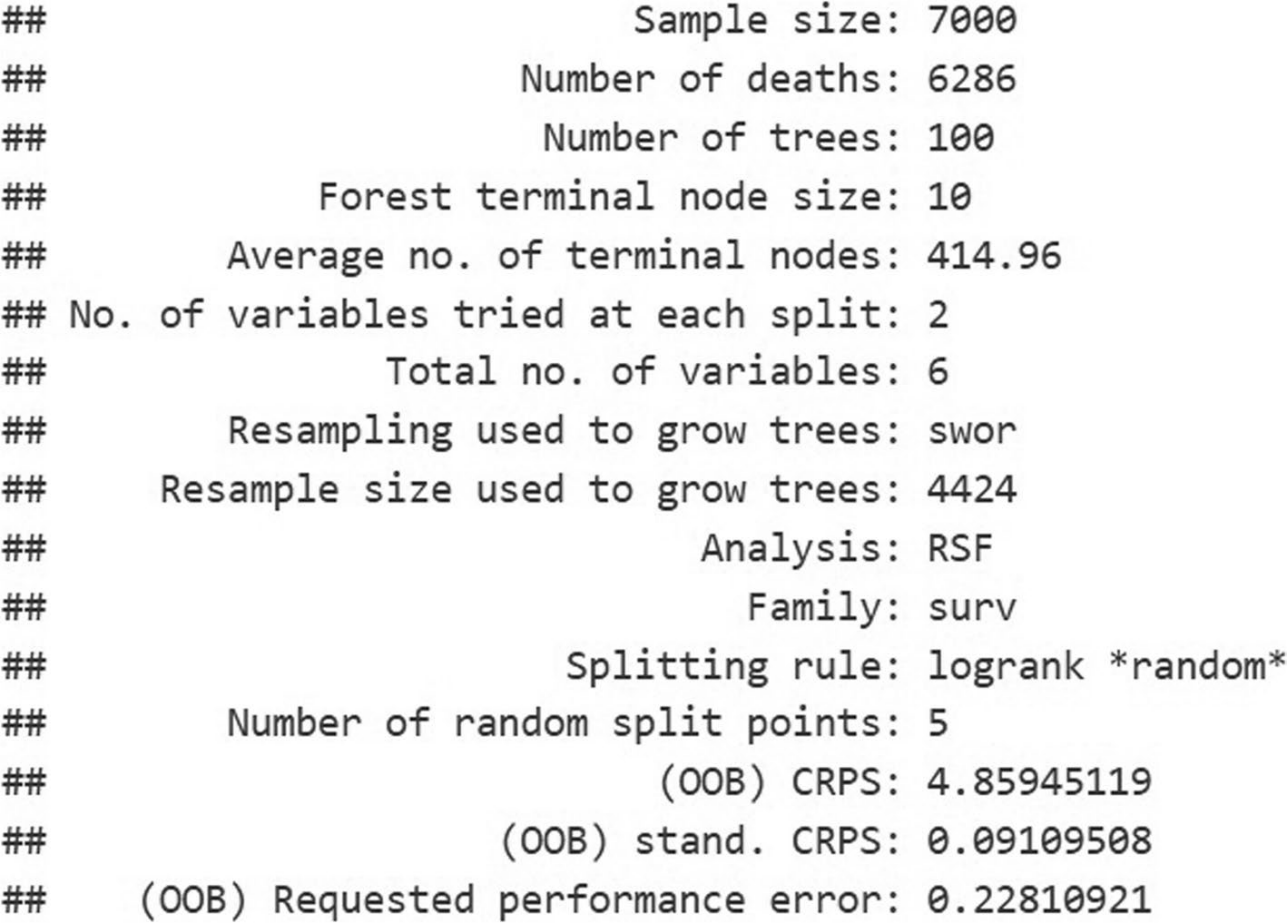
Output of the RSF model. Summary of key parameters and performance metrics for the Random Survival Forest (RSF) model. The figure highlights the number of trees (100), minimum terminal node size (10), and OOB measures (CRPS, performance error), underscoring the model’s suitability for analyzing complex, censored data in kidney transplantation analysis

The RSF model was fitted to 7000 observations, among which 6286 were documented events. This best-performing configuration, featuring 100 trees (*ntree = 100*), a minimum terminal node size of 10 (*nodesize = 10*), and two variables sampled at each split (*mtry = 2*), achieved strong predictive performance on censored survival data. The OOB Continuous Ranked Probability Score (CRPS) was 4.859, while the standardized OOB CRPS stood at 0.091. Additionally, the OOB performance error was 0.228. These statistics provide a comprehensive overview of the RSF model’s structure and performance, indicating the model’s ability to predict survival outcomes based on the given predictor variables (Fig. 2).

### Step 4: Testing the RSF algorithm

Model validation is crucial for understanding the model's performance [21]. Evaluating the model on the test data which constitutes 30% of the sample provides insights into how well the model is likely to perform in real-world scenarios [17].

In this step, several evaluation metrics are calculated using the test dataset, including the C-index, Brier score, time-dependent AUC, F1 score, accuracy, sensitivity, specificity, positive predictive value, and negative predictive value. The Integrated Brier Score (IBS) evaluates the accuracy of predicted probabilities, while the concordance index (C-index) measures the correct ranking of outcomes [22]. Time-dependent AUC assesses the model's ability to distinguish between different outcomes over time. F1 score is the harmonic mean of precision and recall, providing a balance between the two. Accuracy is the proportion of true results (both true positives and true negatives) among the total number of cases examined. Sensitivity (Recall) is the ability of the model to correctly identify true positives. Specificity is the ability of the model to correctly identify true negatives. Positive predictive value (Precision) is the proportion of positive results that are true positives. Negative predictive value is the proportion of negative results that are true negatives.

The metrics of accuracy, sensitivity, specificity, and positive and negative predictive values can be calculated using the confusionMatrix function from the caret package in R. This function takes the model's predictions and the actual values as inputs, generating a confusion matrix that summarizes the model's performance. From this matrix, the mentioned metrics can be derived, providing a detailed evaluation of the model's ability to correctly classify observations into different categories.

#### Code Block: Calculating the Confusion Matrix

# Load the required library.

library(caret).

# Extract predictions from the RSF model.

predicted_values <—predict(best_model, newdata = test_data)$predicted.

# Extract survival probabilities from the RSF model at a specific time point.

time_point <—60 # Specify the time point of interest.

predicted_probabilities <—predict(best_model, newdata = test_data, type = "prob", times = time_point)$survival.

# Convert survival probabilities to failure probabilities.

failure_probabilities <—1—predicted_probabilities.

# Select the last column of the predicted probabilities (time 60).

test_data$pr_failure <—failure_probabilities[, ncol(failure_probabilities)].

# Convert the predictions to factors.

test_data$pr_failure2 <—ifelse(test_data$pr_failure > 0.6, 1, 0).

test_data$pr_failure2 < -as.factor(test_data$pr_failure2).

test_data$event_occurred_factor < -as.factor(test_data$event_occurred).

# Ensure both factors have the same levels in the same order.

levels(test_data$pr_failure2) <—levels(test_data$event_occurred_factor).

# Calculate the confusion matrix for the new test set.

new_test_confusion < -confusionMatrix(test_data$pr_failure2,test_data$event_occurred_factor, positive = "1").

# Print the Confusion Matrix.

print(new_test_confusion).

**Fig. 3:**
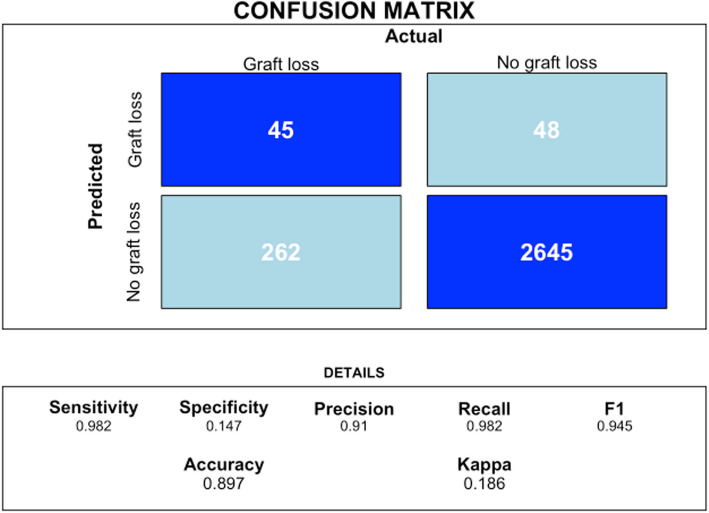
RSF model confusion matrix. The confusion matrix output shows that the model has an accuracy of 89.67%, meaning it correctly classifies 89.67% of the instances. The sensitivity (recall) is high at 98.22%, indicating the model effectively identifies true positives. However, the specificity is low at 14.66%, meaning it struggles to correctly identify true negatives. The positive predictive value (precision) is 90.99%, showing that most positive predictions are correct, while the negative predictive value is 48.39%, indicating less reliability in negative predictions. The balanced accuracy, which averages sensitivity and specificity, is 56.44%, reflecting the model's performance on imbalanced data

RSF model confusion matrix (Fig. 3).

### Step 5: Model validation: Assessing performance of RSF algorithm

The C-index is a key measure for validating survival models, reflecting the model's ability to predict survival times accurately. It indicates how well the model discriminates between the survival outcomes of two subjects based on predictor factors. A C-index of 1 means the model perfectly predicts higher survival for subjects with lower risk, while a C-index of 0.5 indicates random prediction [3]. To calculate the C-index, the proportion of concordant pairs (where the model correctly predicts shorter survival for higher-risk subjects) is divided by the total number of pairs [23]. After training the RSF model, calculate the C-index using the **survcomp** package. This package requires the predictions from the RSF model, along with the actual survival times and the event indicator. The C-index is then calculated using the function **concordance.index(predicted_values, pbc$time, pbc$status). **The R code used is as follows:

#### Code Block: Calculating the concordance index (C-index)

# Load the required library.

library(survcomp).

# Make predictions on the test set.

pred <—predict(rsf_model, newdata = test_data).

# Calculate the concordance index.

c_index <—concordance.index(pred$predicted, test_data$time_to_event,

test_data$event_occurred).

# Print the C-index.

print(paste("Índice C:", c_index$c.index)).

The output of the C-index provides a measure of the predictive discrimination of the RSF model. The C-index value is 0.774, which indicates that the model has a good ability to discriminate between patients who experience the event (graft loss) and those who do not.

To calculate the time-dependent Area Under the Curve (AUC) for our predictive model we used the **risksetROC** function from the **risksetROC** package in R. This analysis was conducted to evaluate the model's discriminatory power over different time points.

#### Code Block: Calculating the time-dependent Area Under the Curve (AUC)

# Load the required library.

library(**risksetROC**).

# Calculate the time-dependent AUC.

time_auc <—risksetROC(

Stime = test_data$time_to_event,

status = test_data$event_occurred,

marker = predicted_values,

predict.time = seq(1, 60, by = 0.5).

)

An AUC of 0.709 indicates that the model has a 70.9% chance of correctly distinguishing between positive and negative classes. This value suggests that the model has a fair level of discrimination ability.

The following code calculates the IBS for the RSF model using the pec package in R. The IBS is a measure of the accuracy of probabilistic predictions, where a lower score indicates better predictive performance. The pec function is used to compute the IBS by comparing the predicted survival probabilities from the RSF model to the actual survival outcomes in the test dataset. The formula argument specifies the survival model, with *Surv(time_to_event, event_occurred)* representing the survival outcome and the predictor variables including age, BMI, diabetes, hypertension, donor age, and cold ischemia time. The times argument defines the time points at which the IBS is calculated, ranging from 0 to the maximum time-to-event value in the test dataset. Finally, the IBS is printed to the console using the print function. The following code demonstrates the process:

#### Code Block: Calculating the Integrated Brier Score (IBS)

# Load the required library.

library(pec).

# Calculate the Integrated Brier Score (IBS).

brier_score <—pec(object = rsf_model,

formula = *Surv(time_to_event, event_occurred)* ~ age + bmi + diabetes + hypertension + donor_age + cold_ischemia_time,

data = test_data).

# Print the Brier Score.

print(brier_score).

The IBS is a metric used to assess the accuracy of probabilistic predictions over a specified time interval, making it particularly valuable in survival analysis. It evaluates the discrepancy between prognostic probabilities and observed outcomes. The interpretation of the IBS is more favorable when the value is closer to 0, indicating higher predictive accuracy. Values greater than 1 suggest that the predictions may be no better than random chance. In this case, the IBS is calculated for the time interval from 0 to 60 months. The output provides the IBS for both the reference model and the RSF model.

The reference model has an IBS of 0.121, while the RSF model has an IBS of 0.090. The lower IBS value for the RSF model indicates that it has slightly better predictive accuracy compared to the reference model over the specified time interval.

The code calculates and plots the calibration curve for the RSF model using the pec package in R. The calibration curve is used to assess the agreement between predicted probabilities and observed probabilities of the event occurring. The pec function computes the calibration curve by comparing the predicted survival probabilities from the RSF model to the actual survival outcomes in the test dataset. The formula argument specifies the survival model, with *Surv(time_to_event, event_occurred)* representing the survival outcome and the predictor variable. The times argument defines the time points at which the calibration curve is calculated, ranging from 0 to the maximum time-to-event value in the test dataset. The splitMethod argument specifies the resampling method used, in this case, “Boot632plus”. Finally, the calibration curve is plotted using the plot function, with the x-axis representing the predicted probabilities and the y-axis representing the observed probabilities. This plot provides a visual assessment of the model’s calibration. The following R code demonstrates this process:

#### Code Block: Calibration curve

# Load the required library.

library(pec).

# Calculate the calibration curve.

calibration_curve <—pec(

object = rsf_model,

formula = *Surv(time_to_event, event_occurred)* ~ age + bmi + diabetes + 

hypertension + donor_age + cold_ischemia_time,

data = test_data,

times = seq(0, max(test_data$time_to_event), by = 10),

splitMethod = "Boot632plus").

# Plot the calibration curve.

plot(calibration_curve,

xlab = "Predicted Probability",

ylab = "Observed Probability",

main = "Calibration Curve").

**Fig. 4:**
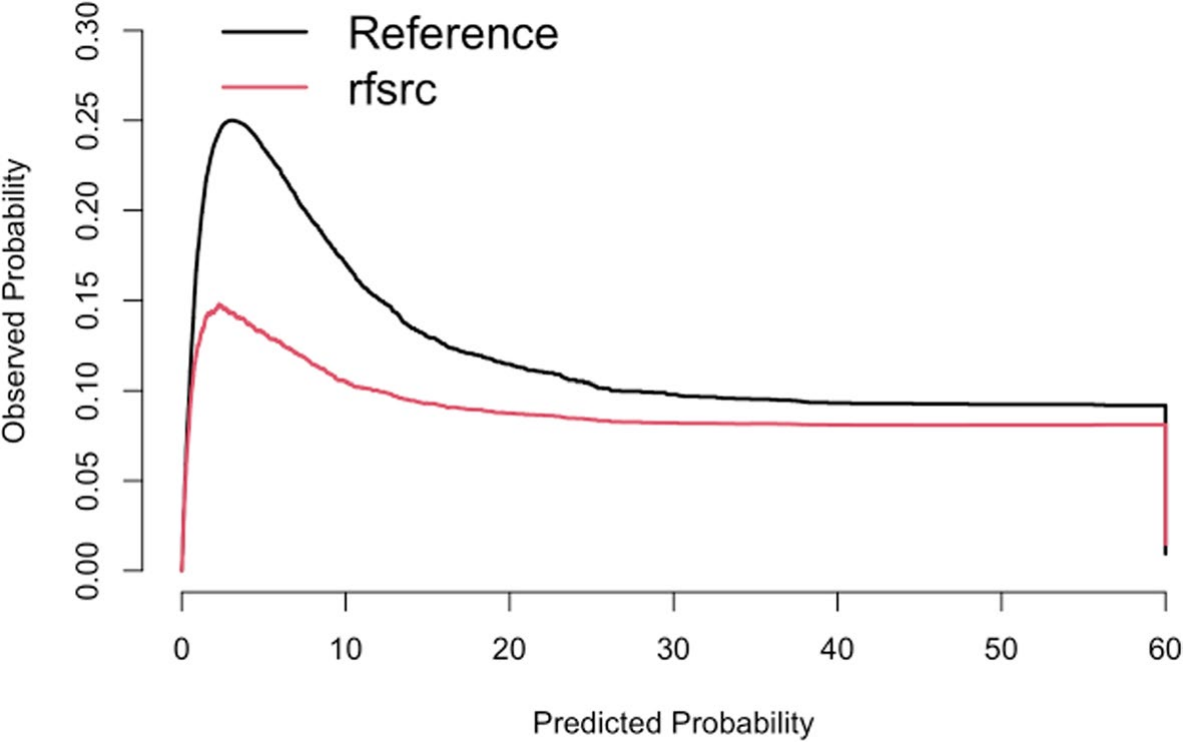
Calibration curve. Calibration curve illustrates the agreement between predicted probabilities and observed probabilities for the RSF model. The black line represents perfect calibration, while the red line shows the model's performance. Deviations from the black line indicate areas of over- or underestimation in predicted probabilities

The calibration curve presented in Fig. 4 compares the predicted probabilities generated by the** rfsrc** model against the observed probabilities. The x-axis represents the predicted probabilities, while the y-axis denotes the observed probabilities. The black line, labeled “Reference,” indicates the ideal scenario where predicted probabilities perfectly match the observed probabilities. The red line represents the performance of the **rfsrc** model.

Finally, we performed a decision curve analysis (DCA) to evaluate the clinical utility of our predictive model using the rmda package in R. The DCA was conducted by applying the decision_curve function to our test dataset, with the outcome variable event_occurred and the predictor pr_failure. The analysis was performed across a range of threshold probabilities from 0 to 1, incremented by 0.01, under an "opt-in" policy framework. To ensure robustness, we employed 100 bootstrap resamples.

#### Code Block: Decision curve analysis

# Load the required library.

library(rmda).

# Perform decision curve analysis using rmda.

dca_results <—decision_curve(event_occurred ~ pr_failure,

data = test_data,

thresholds = seq(0, 1, by = 0.01),

policy = "opt-in",

bootstraps = 100).

# Generate the decision curve plot.

**Fig. 5:**
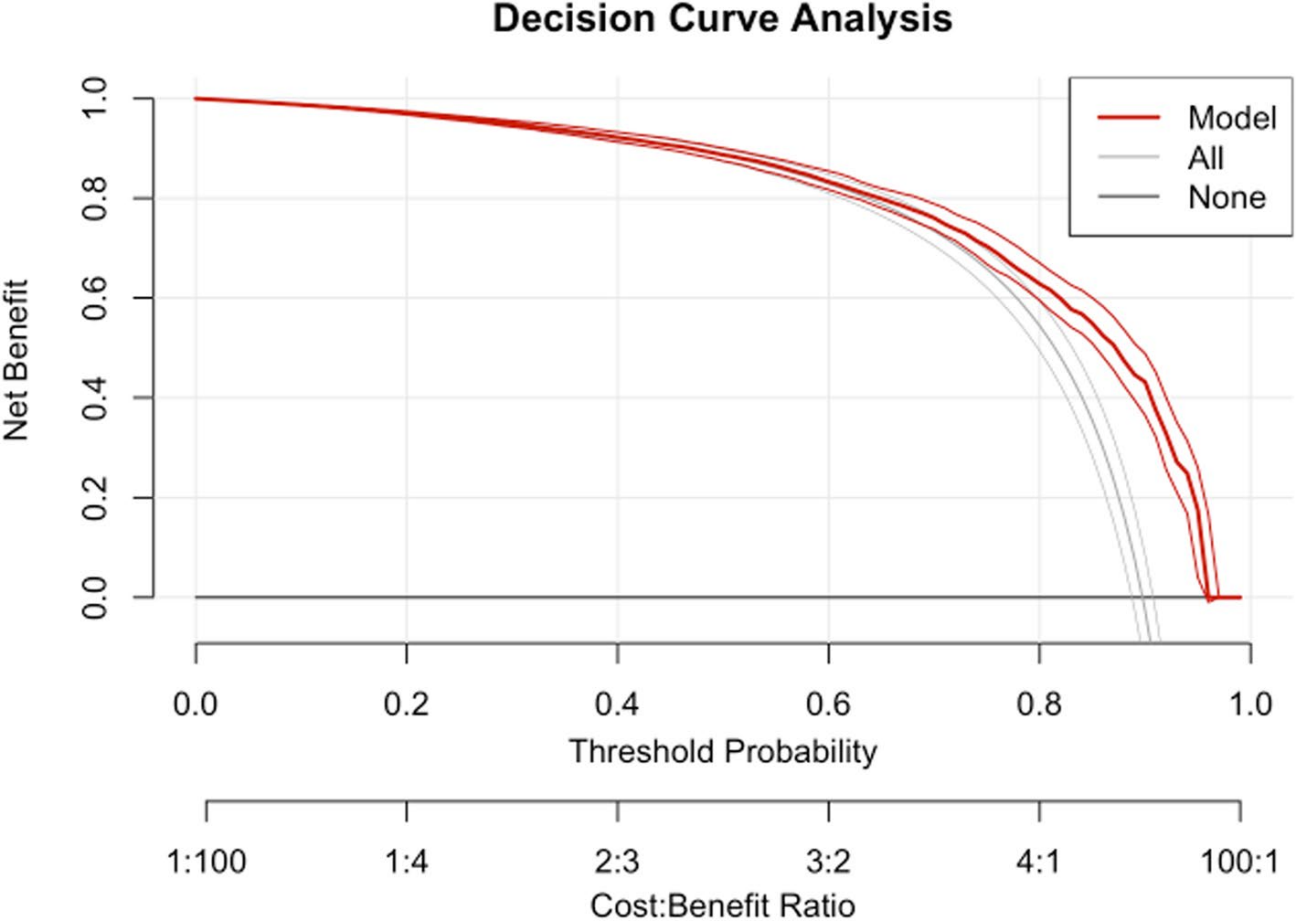
Decision curve analysis. The decision curve plot illustrates the net benefit of using the predictive model across various threshold probabilities. The model curve indicates the net benefit of the predictive model at different threshold probabilities. The model curve consistently lies above the "Treat All" and "Treat None" curves, suggesting that the model provides a higher net benefit than either treating all subjects or treating none. The threshold probability at which the model curve reaches its peak net benefit can be considered the optimal threshold for decision-making. This point represents the maximum clinical utility of the model. By comparing the model curve with the "Treat All" and "Treat None" curves, we can assess the model's improvement over these baseline strategies. The model demonstrates a significant net benefit over both baselines, indicating its potential value in clinical practice

plot_decision_curve(dca_results, curve.names = "Model",

xlab = "Threshold Probability", ylab = "Net Benefit",

main = "Decision Curve Analysis") (Fig. 5).

## Decision curve analysis

### Step 6: Plotting variable importance

The code demonstrates how to visualize the importance of variables in an RSF model using the **ggplot2** package in R. The process begins by creating the **importance_df** data frame to store variable names **Feature** and their corresponding importance scores **Importance**. This data frame is then sorted in descending order of importance. Finally, the **ggplot2** package is used to create a bar chart, where the x-axis represents the variables, and the y-axis shows their importance scores. This visualization highlights the relative contribution of each variable to the model's predictions.

#### Code Block: Variable importance plot

# Load the required library.

library(ggplot2).

# Create a data frame with variable importance.

importance_df <—data.frame(Feature = names(rsf_model$importance), Importance = rsf_model$importance).

importance_df <—importance_df[order(-importance_df$Importance),]

# Plot the variable importance.

ggplot(importance_df, aes(x = reorder(Feature, Importance), y = Importance)) + 

geom_bar(stat = "identity") + 

coord_flip() + 

xlab("Variables") + 

ylab("Importance") + 

ggtitle("Variable Importance") + 

theme_minimal().

**Fig. 6:**
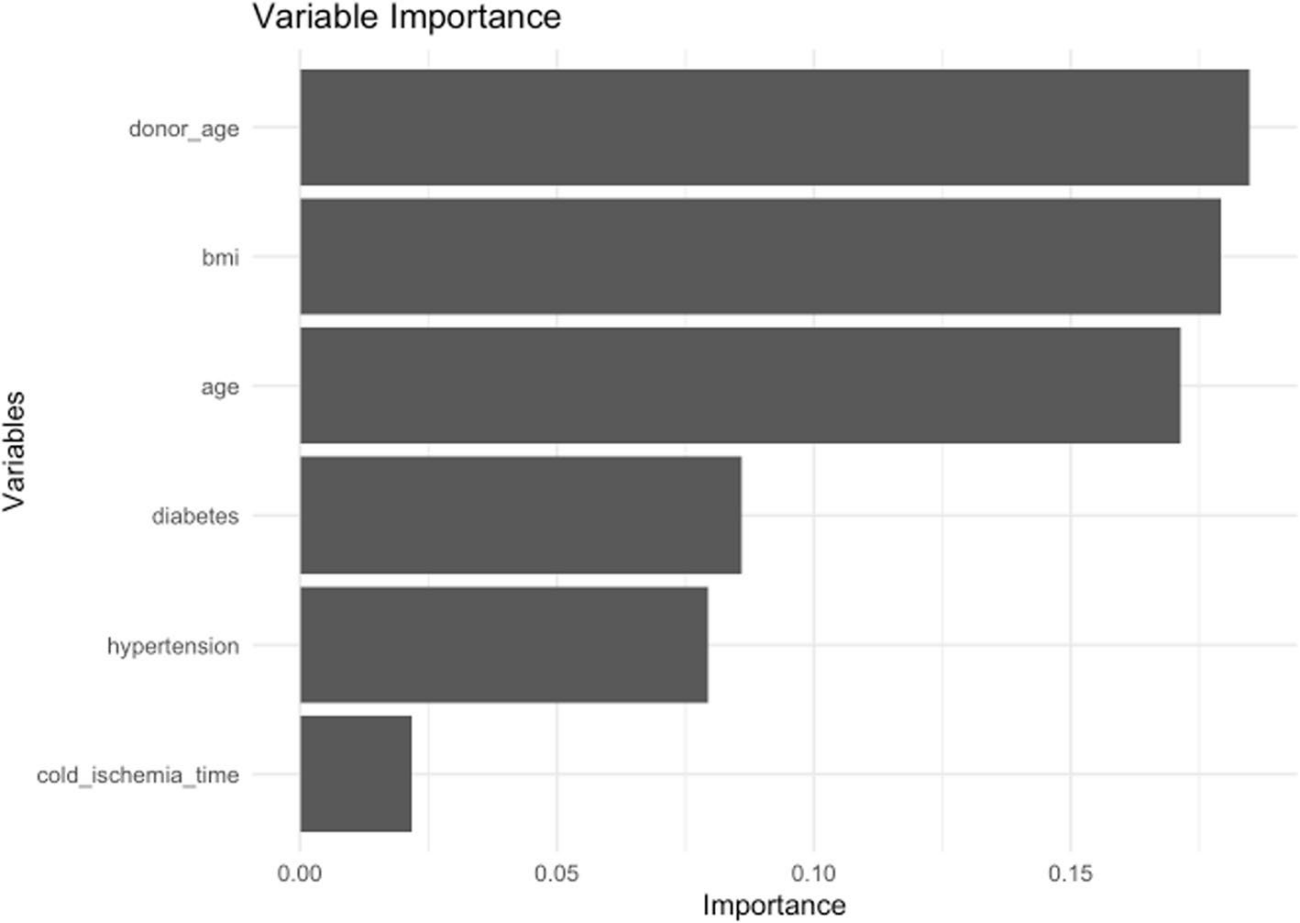
Bar chart of variable importance. Variable importance plot from the RSF model, ranking donor age, recipient body mass index (BMI), and recipient age as the most significant predictors. Additional influential factors include diabetes, hypertension, and cold ischemia time, emphasizing their role in graft survival prediction

The variables are ranked based on their importance, with ‘donor_age’ being the most influential, followed by ‘bmi’, ‘age’, ‘diabetes’, and ‘hypertension’. The x-axis represents the importance score, ranging from 0 to 0.15. This visualization highlights which factors have the greatest impact on the model’s outcomes, providing valuable insights for understanding the underlying data (Fig. 6).

### Step 7. Comparison to Cox Hazard Model

The RSF model will be compared with a Cox Proportional Hazards regression model to evaluate their predictive performance in survival analysis. This comparison is essential because the Cox model, a widely used method in survival analysis, assumes proportional hazards and linear relationships between predictors and the hazard function. In contrast, the RSF model is a non-parametric approach that can capture complex interactions and non-linear relationships without such assumptions.

The process begins by fitting a Cox model using survival data and predictors with the coxph() function. The model results, including coefficients and hazard ratios, are extracted using summary(cox_model) to assess the influence of each variable. 

Then, the C-index is calculated for both models to evaluate the discrimination of the prediction with the actual survival times using the concordance.index()function.

#### Code Block: Cox Model Evaluation and RSF Comparison

# Cox Proportional Hazards Model.

cox_model <—coxph (*Surv(time_to_event, event_occurred)* ~ age + bmi + diabetes + hypertension + donor_age + cold_ischemia_time, data = train_data).

# Summary of the Cox model.

summary(cox_model).

# Predictions on the test set using the Cox model.

cox_pred <—predict(cox_model, newdata = test_data, type = "risk").

# Calculate the C-index for the Cox model.

cox_c_index <—concordance.index(cox_pred, test_data$time_to_event, test_data$event_occurred).

print(paste("C-index for Cox model:", cox_c_index$c.index)).

# Compare C-index of both models.

print(paste("C-index for Random Survival Forest:", c_index$c.index)).

print(paste("C-index for Cox model:", cox_c_index$c.index)).

To calculate main metrics of Cox Hazard Model, we generate a confusion matrix to evaluate the performance by comparing the binary predictions with the actual outcomes **test_data$event_occurred.** The matrix is displayed using the **confusionMatrix **function.

#### Code Block: Cox confusion matrix

# Convert predictions to binary outcomes based on a threshold.

threshold <—median(cox_pred) # You can choose a different threshold.

binary_predictions <—ifelse(cox_pred > threshold, 1, 0).

# Create a confusion matrix using the confusionMatrix function.

conf_matrix <—confusionMatrix(factor(binary_predictions), factor(test_data$event_occurred), positive = "1").

# Print the confusion matrix.

**Fig. 7:**
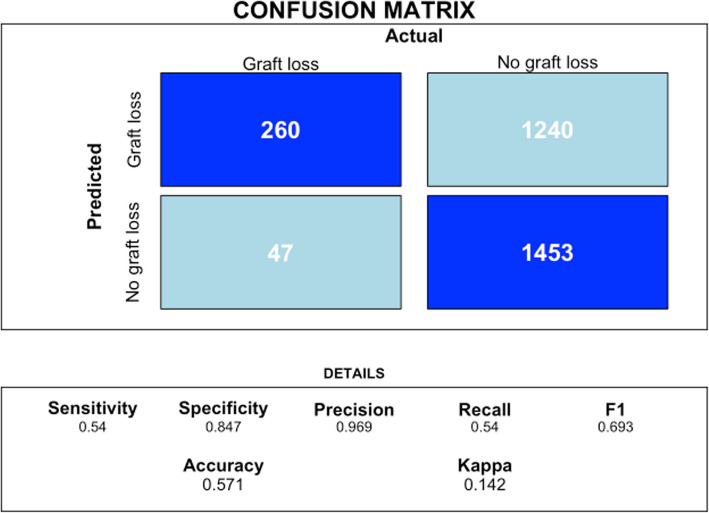
Cox Hazard Model confusion matrix. The RF model excels in accuracy (0.897), sensitivity (0.982), and precision (0.91) compared to the Cox model but has lower specificity (0.147). These results suggest the RF model is highly sensitive in identifying graft loss but generates more false positives

print(conf_matrix) (Fig. 7).

**Ethical Considerations.**

This study was conducted using a simulated database and did not involve real human participants, identifiable human data, or clinical records. Therefore, ethical approval from an institutional review board (IRB) or ethics committee was not required. Likewise, as no human participants were included, the requirement for informed consent was not applicable.

This research adheres to established ethical principles and complies with relevant guidelines for scientific integrity. Although no direct human data were utilized, the study aligns with the ethical standards outlined in the Declaration of Helsinki and the Resolution 8430 of 1993 of the Colombian Ministry of Health, which establishes the ethical norms for health research in Colombia [24, 25]. According to this resolution, studies based exclusively on simulated or publicly available data do not require formal ethical approval.

## Results

The RSF model was evaluated using multiple performance metrics. The OOB CRPS was 4.859, with a standardized OOB CRPS of 0.091 and an OOB requested performance error of 0.228. Additionally, the C-index was 0.774, indicating the model’s ability to discriminate between the evaluated outcomes. When compared to the reference model, the IBS was lower in the RSF model (0.090 vs. 0.121), suggesting a reduced cumulative prediction error.

The time-dependent ROC analysis produced an AUC of 0.709, indicating the model’s capacity to differentiate between events and non-events over time. Furthermore, calibration analysis (Fig. 4) showed that while the reference model performed better for shorter survival times, the RSF model provided more accurate predictions for longer survival times.

In the confusion matrix analysis, the RSF model achieved a sensitivity of 0.982, correctly identifying 98.2% of graft loss cases. However, its specificity was 0.147, indicating a higher rate of false positives. The model had a precision of 0.91, with 91% of predicted graft loss cases being correct, and an F1 score of 0.945, reflecting the balance between precision and recall. The overall accuracy was 0.897, while the Kappa statistic was 0.186, suggesting low agreement beyond chance, potentially due to class imbalance.

Finally, variable importance analysis identified donor age, BMI, and recipient age as the three most influential risk factors for predicting graft loss.

## Discussion

In this study, we conduct a step-by-step review of the construction of an RSF model for a population derived from a public database of kidney transplants and loss of graft in time. The aim is to explore ML methods within actual medical context. The method we employed is highly reproducible and applicable. This study is intended to serve as a foundation for future analyses, whether in the field of transplantation or other areas of clinical interest, in which the primary outcome is the time to event, or survival.

When conducting this statistical analysis, we identified several key aspects of working with a RSF model. It is crucial to use partitioning methods that involve the entire dataset, such as bootstrapping or cross-validation. Hyperparameter tuning must be performed meticulously to ensure the most accurate and reliable results, consistent with the required statistical power. In this context, the number of trees and nodes is particularly important for the model's execution.

Moreover, the model’s internal validity was assessed using the C-index, which must be above 0.5 to demonstrate statistical significance in survival outcomes. In this case, the C-index was 0.774, and the IBS was lower in the RSF model (0.090 vs. 0.047 in the reference model), indicating better performance while also reducing the risk of overfitting.

Likewise, when evaluating previous publications, it was found on the use of similar methodologies in health studies. A 2022 systematic review of ML techniques in lung transplants, which analyzed 16 studies. It found that 25% of the studies used Random Forest models to predict outcomes such as acute disease events, survival rates, recipient-donor matching, rejection, and quality of life [4]. Another 2022 systematic review showed that patients selected for heart transplantation using deep neural networks experienced significant reductions in waitlist mortality and increased post-transplant survival times [26]. A 2023 review on liver transplants explored the use of different ML models at five key stages. These included prioritizing candidates in the pre-transplant phase using MELD and MELD-Na scores with Random Forest models, assessing graft allocation and steatosis with logistic regression, predicting 30-day post-transplant graft failure with artificial neural networks, forecasting patient outcomes and survival with RSF and the C-index, and evaluating the risk of post-transplant comorbidities and complications using gradient boosting machines [27].

The methodology employed in this study, utilizing RSF, underscores its practical application in healthcare by effectively managing censored data and high-dimensional variables [22, 28], which are common in medical datasets. This model's flexibility in handling non-linear relationships and high-dimensional data offers a significant advantage over traditional methods like the Cox proportional hazards model. Moving forward, the practical application of RSF and similar ML methods in healthcare can be expanded to various domains, including personalized medicine and real-time decision-making, potentially improving patient outcomes through more accurate and tailored predictions [29]. Future research could focus on refining the RSF approach by integrating additional clinical variables and testing its applicability across different organ transplant databases. Additionally, exploring hybrid models that combine RSF with other ML techniques may further enhance predictive performance and generalizability in diverse clinical settings.

Additionally, the meta-analysis by Ravindhran et al. [5] highlights the application of ML models, including RSF, in predicting kidney graft survival using real-world clinical data from large registries (5). Their findings demonstrate superior predictive accuracy of ML models, with an AUC-ROC of 0.82, compared to traditional statistical methods. While this study uses simulated data to control variability and ensure reproducibility, future validation with clinical datasets, as emphasized by the authors, is essential to enhance the model's generalizability and clinical relevance.

Also, Fig. 4 shows the calibration curve of the RSF model, with strong alignment to the reference line at lower predicted probabilities, indicating reliable performance for low-risk patients. Slight deviations at higher probabilities suggest minor over- or underestimations, highlighting areas for improvement. The model demonstrates robust calibration, particularly for low to moderate-risk predictions. The application of the RSF model in predicting kidney graft loss provides actionable insights that could significantly enhance clinical decision-making. By identifying key variables such as donor age, recipient BMI, and recipient age as the most critical predictors, the model enables clinicians to better stratify patient risk and tailor post-transplant care. Additionally, the superior performance metrics of the RSF model compared to traditional methods, such as the Cox proportional hazards model, suggest its potential for broader adoption in clinical practice. Future implementations of this model could facilitate personalized treatment plans and optimize donor-recipient matching, ultimately improving graft survival rates and patient quality of life.

The step-by-step guide provided in this study serves not only as a practical framework for implementing the RSF model but also as a valuable educational tool for researchers and clinicians. By offering detailed instructions and reproducible code, this guide complements existing research by making advanced ML techniques more accessible to non-expert audiences. Moreover, its educational value lies in enabling clinicians to better understand the methodological foundations of predictive models, thereby fostering their integration into clinical workflows and research settings. Recent advancements in transplantation highlight hybrid methods, such as combining RSF with deep learning, to enhance predictive accuracy and robustness. While this study demonstrates RSF’s utility using simulated data, the findings may not fully generalize to real-world datasets with greater variability. Future research should focus on validating the model with clinical data to ensure its reliability and facilitate its integration into clinical workflows, improving decision-making and patient outcomes.

Although the RSF model has several strengths over classical methods, it also has some limitations due to the complexity of its analysis. These include higher computational requirements, less intuitive interpretation, and reduced predictive accuracy in small samples [30]. Additionally, it has been observed that, because RSF does not assume proportionality of variables, it may overestimate their effects over time [31].

In this study, we used simulated data to evaluate the RSF model's performance under controlled conditions. While this approach provided consistency and eliminated noise or biases common in real-world datasets, it inherently limits the clinical relevance of our findings. Simulated data may not accurately capture the true distribution or interrelationships of variables observed in actual kidney transplant populations, nor do they reflect causal relationships between predictors and graft loss. Because variable values in the simulation were generated without explicitly modeling causality, the RSF model's applicability as a clinical decision-making tool is limited. Instead, it should be regarded as an educational tool to demonstrate the potential of advanced machine learning methods in survival analysis.

Future research should validate these findings using real-world clinical data to evaluate the model’s robustness in practical applications. Additionally, incorporating causality into the simulation process could enhance the educational utility and provide insights into how predictor-outcome relationships evolve over time. In our study, censoring was administrative. All subjects were followed until the study's end, with those still alive right-censored. This method ensured accurate survival analysis by reflecting the study's duration and participants' status at the conclusion. Right-censoring maintained the integrity of our survival estimates, providing a clear understanding of outcomes within the defined timeframe.

## Conclusion

In conclusion, this study highlights the applicability and reproducibility of the RSF model in analyzing kidney transplant data and graft loss over time. By meticulously selecting partitioning methods and hyperparameter tuning, we achieved robust internal validation, evidenced by a C-index of 0.7 and an IBS of 0.046, both outperforming the reference model. The literature review underscores the increasing use of ML methodologies, like RSF, in transplantation outcomes. Despite some limitations, such as computational demands and interpretative complexity, RSF offers significant advantages in managing censored data and high-dimensional variables. Future research should focus on integrating additional clinical variables and exploring hybrid models to enhance predictive accuracy and applicability in diverse clinical contexts.

In addition to its methodological contributions, this work serves as a practical and educational guide for applying RSF models. The emphasis on clarity, reproducibility, and practical examples makes it a valuable resource for both researchers and clinicians aiming to integrate machine learning techniques into survival analysis.

## Clinical trial number

Not applicable.

## Supplementary Information

Supplementary Material 1

Supplementary Material 2

Supplementary Material 3

## Data Availability

The code for the dataset is available in Supplementary Material 1, the generated dataset and the R script used for the analysis are provided in Supplementary Material 2 and 3.

## Abbreviations

AI: Artificial Intelligence
AUC: Area Under the Curve
BMI: Body Mass Index
CRPS: Continuous Ranked Probability Score
DCA: Decision Curve Analysis
IBS: The Integrated Brier Score
MELD: Model for End-stage Liver Disease
ML: Machine Learning
OOB: Out-Of-Bag
RSF: Random Survival Forest
