# Supplementary material for: Survival analysis using machine learning in transplantation: a practical introduction
Source: BMC Med Inform Decis Mak. 2025 Mar 21;25:141. doi: 10.1186/s12911-025-02951-7 (PMC11929298; doi:10.1186/s12911-025-02951-7)
Supplement: Supplementary file 3 — Supplementary Material 3 [file 12911_2025_2951_MOESM3_ESM.html]

Using Random Survival Forest to predict graft loss in kidney Transplantation


# Using Random Survival Forest to predict graft loss in kidney Transplantation

# **Load necesary libraries**

```
# Load necessary libraries
#install.packages("readxl")
library(readxl)
#install.packages("randomForestSRC")
library(randomForestSRC)
```

```
## 
##  randomForestSRC 3.3.3 
##  
##  Type rfsrc.news() to see new features, changes, and bug fixes. 
##
```

```
#install.packages("survival")
library(survival)
#install.packages("caret")
library(caret)
```

```
## Loading required package: ggplot2
```

```
## Loading required package: lattice
```

```
## 
## Attaching package: 'caret'
```

```
## The following object is masked from 'package:survival':
## 
##     cluster
```

```
#install.packages("survcomp")
library(survcomp)
```

```
## Loading required package: prodlim
```

```
library(pec)
```

```
## 
## Attaching package: 'pec'
```

```
## The following object is masked from 'package:caret':
## 
##     R2
```

```
#install.packages("timeROC")
library(timeROC)
#install.packages("dcurves")
library(dcurves)
#install.packages("ggplot2")
library(ggplot2)
#install.packages("dplyr")
library(dplyr)
```

```
## 
## Attaching package: 'dplyr'
```

```
## The following objects are masked from 'package:stats':
## 
##     filter, lag
```

```
## The following objects are masked from 'package:base':
## 
##     intersect, setdiff, setequal, union
```

```
#install.packages("rmda")
library(rmda)
```

# **Importing and preparing the dataset**

```
# Import the dataset
transplant_database <- read_excel("transplant_database_v04.xlsx")
data <- transplant_database


data$diabetes_factor<-as.factor(data$diabetes)
data$hypertension_factor<-as.factor(data$hypertension)
data$donor_age_factor <-as.factor(data$ donor_age)
data$event_occurred_factor <-as.factor(data$event_occurred)


# Summarize the dataset
summary(data)
```

```
##       age             bmi            diabetes       hypertension   
##  Min.   :10.78   Min.   : 5.718   Min.   :0.0000   Min.   :0.0000  
##  1st Qu.:43.27   1st Qu.:21.690   1st Qu.:0.0000   1st Qu.:0.0000  
##  Median :49.97   Median :25.079   Median :0.0000   Median :0.0000  
##  Mean   :49.98   Mean   :25.068   Mean   :0.1943   Mean   :0.2981  
##  3rd Qu.:56.71   3rd Qu.:28.469   3rd Qu.:0.0000   3rd Qu.:1.0000  
##  Max.   :89.26   Max.   :47.395   Max.   :1.0000   Max.   :1.0000  
##    donor_age      cold_ischemia_time time_to_event      event_occurred  
##  Min.   :0.0000   Min.   : 1.409     Min.   : 0.00027   Min.   :0.0000  
##  1st Qu.:0.0000   1st Qu.: 8.673     1st Qu.: 1.12989   1st Qu.:1.0000  
##  Median :0.0000   Median :10.018     Median : 3.14439   Median :1.0000  
##  Mean   :0.4039   Mean   :10.006     Mean   :10.36614   Mean   :0.8979  
##  3rd Qu.:1.0000   3rd Qu.:11.343     3rd Qu.: 8.57180   3rd Qu.:1.0000  
##  Max.   :1.0000   Max.   :17.491     Max.   :60.00000   Max.   :1.0000  
##  diabetes_factor hypertension_factor donor_age_factor event_occurred_factor
##  0:8057          0:7019              0:5961           0:1021               
##  1:1943          1:2981              1:4039           1:8979               
##                                                                            
##                                                                            
##                                                                            
##
```

# **Data splitting**

```
# Set seed for reproducibility
set.seed(42)

# Split the data into training and testing sets
n <- nrow(data)
train_indices <- sample(1:n, size = 0.7 * n)
train_data <- data[train_indices, ]
test_data <- data[-train_indices, ]
```

# **Training and validating the RSF algorithm**

```
# Define the cross-validation method
cv_control <- trainControl(method = "cv", number = 5)

# Define the grid of hyperparameters to tune
tune_grid <- expand.grid(ntree = c(50, 100),
                         nodesize = c(10, 15),
                         mtry = c(2, 3))

# Function to train the model with specific hyperparameters
train_rsf <- function(ntree, nodesize, mtry) {
  rfsrc(Surv(time_to_event, event_occurred) ~ age + bmi + diabetes + hypertension + 
        donor_age + cold_ischemia_time,
        data = train_data,
        ntree = ntree,
        nodesize = nodesize,
        mtry = mtry,
        nsplit = 5,
        importance = TRUE,
        seed = 42)
}

# Perform cross-validation manually
cv_results <- lapply(1:nrow(tune_grid), function(i) {
  params <- tune_grid[i, ]
  model <- train_rsf(params$ntree, params$nodesize, params$mtry)
  pred <- predict(model, newdata = test_data)
  c_index <- concordance.index(pred$predicted, test_data$time_to_event, test_data$event_occurred)
  list(model = model, c_index = c_index$c.index)
})

# Select the best model based on C-index
best_model <- cv_results[[which.max(sapply(cv_results, function(x) x$c_index))]]$model

print(best_model)
```

```
##                          Sample size: 7000
##                     Number of deaths: 6286
##                      Number of trees: 100
##            Forest terminal node size: 10
##        Average no. of terminal nodes: 414.96
## No. of variables tried at each split: 2
##               Total no. of variables: 6
##        Resampling used to grow trees: swor
##     Resample size used to grow trees: 4424
##                             Analysis: RSF
##                               Family: surv
##                       Splitting rule: logrank *random*
##        Number of random split points: 5
##                           (OOB) CRPS: 4.85945119
##                    (OOB) stand. CRPS: 0.09109508
##    (OOB) Requested performance error: 0.22810921
```

```
best_model <- 
  rfsrc(Surv(time_to_event, event_occurred) ~ age + bmi + diabetes + hypertension + 
        donor_age + cold_ischemia_time,
        data = train_data,
        ntree = 100,
        nodesize = 10,
        mtry = 2,
        nsplit = 5,
        importance =TRUE ,
        seed = 42)
```

# **Model validation on the test set**

```
# Extract predictions from the RSF model
predicted_values <- predict(best_model, newdata = test_data)$predicted

# Extract survival probabilities from the RSF model at a specific time point
time_point <- 60 # Specify the time point of interest
predicted_probabilities <- predict(best_model, newdata = test_data, type = "prob", times = time_point)$survival

# Convert survival probabilities to failure probabilities
failure_probabilities <- 1 - predicted_probabilities

# Select the last column of the predicted probabilities
test_data$pr_failure <- failure_probabilities[, ncol(failure_probabilities)]

test_data$pr_survival <- predicted_probabilities[, ncol(predicted_probabilities)]


# Convert the predictions to factors
test_data$pr_failure2 <- ifelse(test_data$pr_failure > 0.6, 1, 0)
test_data$pr_failure2<-as.factor(test_data$pr_failure2)
test_data$event_occurred_factor<-as.factor(test_data$event_occurred)

# Ensure both factors have the same levels in the same order
levels(test_data$pr_failure2) <- levels(test_data$event_occurred_factor)

# Calculate the confusion matrix for the new test set
new_test_confusion <- confusionMatrix(test_data$pr_failure2, test_data$event_occurred_factor, positive = "1")
print(new_test_confusion)
```

```
## Confusion Matrix and Statistics
## 
##           Reference
## Prediction    0    1
##          0   45   48
##          1  262 2645
##                                           
##                Accuracy : 0.8967          
##                  95% CI : (0.8852, 0.9073)
##     No Information Rate : 0.8977          
##     P-Value [Acc > NIR] : 0.5865          
##                                           
##                   Kappa : 0.1863          
##                                           
##  Mcnemar's Test P-Value : <2e-16          
##                                           
##             Sensitivity : 0.9822          
##             Specificity : 0.1466          
##          Pos Pred Value : 0.9099          
##          Neg Pred Value : 0.4839          
##              Prevalence : 0.8977          
##          Detection Rate : 0.8817          
##    Detection Prevalence : 0.9690          
##       Balanced Accuracy : 0.5644          
##                                           
##        'Positive' Class : 1               
##
```

# **Model performance**

```
draw_confusion_matrix <- function(cm) {

  layout(matrix(c(1,1,2)))
  par(mar=c(2,2,2,2))
  plot(c(100, 345), c(300, 450), type = "n", xlab="", ylab="", xaxt='n', yaxt='n')
  title('CONFUSION MATRIX', cex.main=2)

  # create the matrix 
  rect(150, 430, 240, 370, col= "blue")
  text(195, 435, 'Graft loss', cex=1.5)
  rect(250, 430, 340, 370, col="lightblue")
  text(295, 435, 'No graft loss', cex=1.5)
  text(125, 370, 'Predicted', cex=1.6, srt=90, font=2)
  text(245, 450, 'Actual', cex=1.6, font=2)
  rect(150, 305, 240, 365, col="lightblue")
  rect(250, 305, 340, 365, col="blue")
  text(140, 400, 'Graft loss', cex=1.5, srt=90)
  text(140, 335, 'No graft loss', cex=1.5, srt=90)

  # add in the cm results 
  res <- as.numeric(cm$table)
  text(195, 400, res[1], cex=2, font=2, col='white')
  text(195, 335, res[2], cex=2, font=2, col='white')
  text(295, 400, res[3], cex=2, font=2, col='white')
  text(295, 335, res[4], cex=2, font=2, col='white')

  # add in the specifics 
  plot(c(100, 0), c(100, 0), type = "n", xlab="", ylab="", main = "DETAILS", xaxt='n', yaxt='n')
  text(10, 85, names(cm$byClass[1]), cex=1.5, font=2)
  text(10, 70, round(as.numeric(cm$byClass[1]), 3), cex=1.2)
  text(30, 85, names(cm$byClass[2]), cex=1.5, font=2)
  text(30, 70, round(as.numeric(cm$byClass[2]), 3), cex=1.2)
  text(50, 85, names(cm$byClass[5]), cex=1.5, font=2)
  text(50, 70, round(as.numeric(cm$byClass[5]), 3), cex=1.2)
  text(70, 85, names(cm$byClass[6]), cex=1.5, font=2)
  text(70, 70, round(as.numeric(cm$byClass[6]), 3), cex=1.2)
  text(90, 85, names(cm$byClass[7]), cex=1.5, font=2)
  text(90, 70, round(as.numeric(cm$byClass[7]), 3), cex=1.2)

  # add in the accuracy information 
  text(30, 35, names(cm$overall[1]), cex=1.5, font=2)
  text(30, 20, round(as.numeric(cm$overall[1]), 3), cex=1.4)
  text(70, 35, names(cm$overall[2]), cex=1.5, font=2)
  text(70, 20, round(as.numeric(cm$overall[2]), 3), cex=1.4)
}
```

```
#Confusion matrix 
draw_confusion_matrix(new_test_confusion)
```

```
# Predictions on the test set
pred <- predict(best_model, newdata = test_data)

# Calculate the C-index
c_index <- concordance.index(pred$predicted, test_data$time_to_event, test_data$event_occurred)
print(paste("C-index:", c_index$c.index))
```

```
## [1] "C-index: 0.774271716527063"
```

```
# Calculate the Brier Score
brier_score <- pec(object = best_model, 
                   formula = Surv(time_to_event, event_occurred) ~ age + bmi + diabetes + hypertension + donor_age + cold_ischemia_time, 
                   data = test_data, 
                   times = seq(1, 60, by = 1))
```

```
## Warning in formula.character(object, env = baseenv()): Using formula(x) is deprecated when x is a character vector of length > 1.
##   Consider formula(paste(x, collapse = " ")) instead.
```

```
print(brier_score)
```

```
## 
## Prediction error curves
## 
## Prediction models:
## 
## Reference     rfsrc 
## Reference     rfsrc 
## 
## Right-censored response of a survival model
## 
## No.Observations: 3000 
## 
## Pattern:
##                 Freq
##  event          2693
##  right.censored 307 
## 
## IPCW: cox model
## 
## No data splitting: either apparent or independent test sample performance
## 
## Cumulative prediction error, aka Integrated Brier score  (IBS)
##  aka Cumulative rank probability score
## 
## Range of integration: 0 and time=60 :
## 
## 
## Integrated Brier score (crps):
## 
##           IBS[0;time=60)
## Reference          0.121
## rfsrc              0.090
```

```
# Calculate the calibration curve
 
calibration_curve <- pec(object = best_model,
                          formula = Surv(time_to_event, event_occurred) ~ age + bmi + diabetes + 
                          hypertension + donor_age + cold_ischemia_time,
                          data = test_data,
                          times = seq(0, max(test_data$time_to_event), by = 10),
                          splitMethod = "Boot632plus")
```

```
## Warning in formula.character(object, env = baseenv()): Using formula(x) is deprecated when x is a character vector of length > 1.
##   Consider formula(paste(x, collapse = " ")) instead.
```

```
## Computing noinformation error using all permutations
```

```
## Split sample loop (B=100)
```

```
## Warning: executing %dopar% sequentially: no parallel backend registered
```

```
## 10
```

```
## 20
```

```
## 30
```

```
## 40
```

```
## 50
```

```
## 60
```

```
## 70
```

```
## 80
```

```
## 90
```

```
## 100
```

```
# Plot the calibration curve
plot(calibration_curve, xlab = "Predicted Probability", 
                        ylab = "Observed Probability", main = "Calibration Curve")
```

```
#Make sure that pred$predicted is a vector to calculate the time-dependent AUC
predicted_values <- as.vector(pred$predicted)

# Calculate the time-dependent AUC
library(risksetROC)
```

```
## Loading required package: MASS
```

```
## 
## Attaching package: 'MASS'
```

```
## The following object is masked from 'package:dplyr':
## 
##     select
```

```
time_auc <- risksetROC(
  Stime = test_data$time_to_event,
  status = test_data$event_occurred,
  marker = predicted_values,
  predict.time = seq(1, 60, by =0.5 )
)
```

```
## Warning in Stime >= Target: longer object length is not a multiple of shorter
## object length
```

```
## Warning in entry <= Target: longer object length is not a multiple of shorter
## object length
```

```
## Warning in Stime == Target: longer object length is not a multiple of shorter
## object length
```

```
 # Print the results
tail(time_auc$AUC, n = 1)
```

```
## [1] 0.710266
```

```
# Perform decision curve analysis using rmda
dca_results <- decision_curve(event_occurred ~ pr_failure, 
                              data = test_data, 
                              thresholds = seq(0, 1, by = 0.01), 
                              policy = "opt-in", 
                              bootstraps = 100)

# Generate the decision curve plot
plot_decision_curve(dca_results, curve.names = "Model", 
                    xlab = "Threshold Probability", ylab = "Net Benefit", 
                    main = "Decision Curve Analysis")
```

```
# Variable importance
importance_values <- best_model$importance
importance_df <- data.frame(Feature = names(importance_values), Importance = importance_values)
importance_df <- importance_df[order(-importance_df$Importance), ]
print(importance_df)
```

```
##                               Feature Importance
## donor_age                   donor_age 0.18482805
## bmi                               bmi 0.17930829
## age                               age 0.17142721
## diabetes                     diabetes 0.08591434
## hypertension             hypertension 0.07939816
## cold_ischemia_time cold_ischemia_time 0.02172622
```

```
# Create the bar plot
ggplot(importance_df, aes(x = reorder(Feature, Importance), y = Importance)) +
  geom_bar(stat = "identity") +
  coord_flip() +
  xlab("Variables") +
  ylab("Importance") +
  ggtitle("Variable Importance") +
  theme_minimal()
```

# **Comparison to a Cox Proportional Hazards Model**

```
# Cox Proportional Hazards Model
cox_model <- coxph(Surv(time_to_event, event_occurred) ~ age + bmi + diabetes + hypertension + 
                   donor_age + cold_ischemia_time, data = train_data)

# Summary of the Cox model
summary(cox_model)
```

```
## Call:
## coxph(formula = Surv(time_to_event, event_occurred) ~ age + bmi + 
##     diabetes + hypertension + donor_age + cold_ischemia_time, 
##     data = train_data)
## 
##   n= 7000, number of events= 6286 
## 
##                        coef exp(coef) se(coef)     z Pr(>|z|)    
## age                0.053180  1.054619 0.001327 40.06   <2e-16 ***
## bmi                0.103150  1.108657 0.002693 38.30   <2e-16 ***
## diabetes           1.052840  2.865778 0.032509 32.39   <2e-16 ***
## hypertension       0.852098  2.344562 0.028003 30.43   <2e-16 ***
## donor_age          1.255337  3.509019 0.027343 45.91   <2e-16 ***
## cold_ischemia_time 0.073422  1.076185 0.006425 11.43   <2e-16 ***
## ---
## Signif. codes:  0 '***' 0.001 '**' 0.01 '*' 0.05 '.' 0.1 ' ' 1
## 
##                    exp(coef) exp(-coef) lower .95 upper .95
## age                    1.055     0.9482     1.052     1.057
## bmi                    1.109     0.9020     1.103     1.115
## diabetes               2.866     0.3489     2.689     3.054
## hypertension           2.345     0.4265     2.219     2.477
## donor_age              3.509     0.2850     3.326     3.702
## cold_ischemia_time     1.076     0.9292     1.063     1.090
## 
## Concordance= 0.782  (se = 0.003 )
## Likelihood ratio test= 4854  on 6 df,   p=<2e-16
## Wald test            = 4912  on 6 df,   p=<2e-16
## Score (logrank) test = 4955  on 6 df,   p=<2e-16
```

```
# Predictions on the test set using the Cox model
cox_pred <- predict(cox_model, newdata = test_data, type = "risk")


# Calculate the C-index for the Cox model
cox_c_index <- concordance.index(cox_pred, test_data$time_to_event, test_data$event_occurred)
print(paste("C-index for Cox model:", cox_c_index$c.index))
```

```
## [1] "C-index for Cox model: 0.784283782044327"
```

```
# Compare C-index of both models
print(paste("C-index for Random Survival Forest:", c_index$c.index))
```

```
## [1] "C-index for Random Survival Forest: 0.774271716527063"
```

```
print(paste("C-index for Cox model:", cox_c_index$c.index))
```

```
## [1] "C-index for Cox model: 0.784283782044327"
```

```
# Convert predictions to binary outcomes based on a threshold
threshold <- median(cox_pred)  # You can choose a different threshold
binary_predictions <- ifelse(cox_pred > threshold, 1, 0)

# Create a confusion matrix using the confusionMatrix function
conf_matrix <- confusionMatrix(factor(binary_predictions), factor(test_data$event_occurred), positive = "1")

# Print the confusion matrix
print(conf_matrix)
```

```
## Confusion Matrix and Statistics
## 
##           Reference
## Prediction    0    1
##          0  260 1240
##          1   47 1453
##                                           
##                Accuracy : 0.571           
##                  95% CI : (0.5531, 0.5888)
##     No Information Rate : 0.8977          
##     P-Value [Acc > NIR] : 1               
##                                           
##                   Kappa : 0.142           
##                                           
##  Mcnemar's Test P-Value : <2e-16          
##                                           
##             Sensitivity : 0.5395          
##             Specificity : 0.8469          
##          Pos Pred Value : 0.9687          
##          Neg Pred Value : 0.1733          
##              Prevalence : 0.8977          
##          Detection Rate : 0.4843          
##    Detection Prevalence : 0.5000          
##       Balanced Accuracy : 0.6932          
##                                           
##        'Positive' Class : 1               
##
```

```
#Confusion matrix 
draw_confusion_matrix(conf_matrix)
```
